# The effect of challenge and threat states on performance: An examination of potential mechanisms

**DOI:** 10.1111/j.1469-8986.2012.01449.x

**Published:** 2012-08-22

**Authors:** Lee J Moore, Samuel J Vine, Mark R Wilson, Paul Freeman

**Affiliations:** College of Life and Environmental Sciences, University of ExeterExeter, UK

**Keywords:** Challenge, Threat, Demand/resource evaluations, Emotions, Kinematics, Quiet eye

## Abstract

Challenge and threat states predict future performance; however, no research has examined their immediate effect on motor task performance. The present study examined the effect of challenge and threat states on golf putting performance and several possible mechanisms. One hundred twenty-seven participants were assigned to a challenge or threat group and performed six putts during which emotions, gaze, putting kinematics, muscle activity, and performance were recorded. Challenge and threat states were successively manipulated via task instructions. The challenge group performed more accurately, reported more favorable emotions, and displayed more effective gaze, putting kinematics, and muscle activity than the threat group. Multiple putting kinematic variables mediated the relationship between group and performance, suggesting that challenge and threat states impact performance at a predominately kinematic level.

Like many other contexts (e.g., surgery, military, aviation), competitive sport is characterized by highly pressurized situations that place individuals under extreme stress. However, research examining the effects of stress on sporting task performance has shown considerable variability, from no effect to either facilitative or debilitative effects (for a review, see Hanton, Neil, & Mellalieu, 2008). This variability is likely caused by the individualistic way in which individuals respond to stress (Cerin, Szabo, Hunt, & Williams, 2000). One theoretical framework that offers a potential explanation for such individual differences in stress response is the biopsychosocial model (BPSM) of challenge and threat (Blascovich, 2008a).

## Challenge and Threat States

According to the BPSM (Blascovich, 2008a), prior to a task, individuals evaluate the demands of the task (demand evaluation) and whether they possess the necessary resources to cope effectively with these demands (resource evaluation). Importantly, these evaluations only occur in motivated performance situations (e.g., exam taking, speech giving, sporting competition) and when individuals are actively engaged in a task, evidenced by increases in heart rate and reductions in cardiac pre-ejection period (Seery, 2011). When an individual evaluates that he or she has sufficient resources to meet the demands of the task, a challenge state occurs. In contrast, when an individual evaluates that he or she does not possess the resources required to meet the demands of the task, a threat state emerges (Seery, 2011). Demand and resource evaluations are not only influenced by whether the individual possesses the skills, knowledge, and abilities to perform well on the task. Indeed, several other factors are proposed to impact both demand and resource evaluations, including psychological and physical danger, familiarity, uncertainty, required effort, and the presence of others (Blascovich, 2008a).

Demand and resource evaluations can occur consciously, unconsciously (i.e., automatically), or both (Blascovich, 2008a). However, most authors argue these evaluations are predominately unconscious and automatic, with an individual arriving at a challenge or threat state without any awareness of the evaluation process (Blascovich & Mendes, 2000; Seery, 2011). Thus, a critical component of the BPSM is that challenge and threat states are best indexed objectively via distinctive patterns of neuroendocrine and cardiovascular responses (Blascovich, 2008a; Seery, 2011). Both challenge and threat states are hypothesized to result in elevated sympathetic–adrenomedullary activation causing the release of catecholamines, while a threat state is also predicted to result in elevated pituitary–adrenocortical activation causing the release of cortisol (Seery, 2011). Consequently, a challenge state is marked by relatively higher cardiac output and lower total peripheral resistance compared to a threat state (Seery, 2011). These cardiovascular markers have been well validated in the literature (for a review, see Blascovich, 2008a).

Empirical and predictive studies in psychology across a range of tasks and contexts have shown that a challenge state facilitates performance whereas a threat state hinders performance (Gildea, Schneider, & Shebilske, 2007; Mendes, Blascovich, Hunter, Lickel, & Jost, 2007; Seery, Weisbuch, Hetenyi, & Blascovich, 2010). For example, Blascovich, Seery, Mugridge, Norris, and Weisbuch (2004) found that baseball and softball players who displayed cardiovascular markers of challenge during a 3-min sport-relevant speech 4 to 6 months prior to the start of the season performed better during the subsequent season than players who displayed markers of threat. To date, no research has examined the immediate effects of challenge and threat states on motor task performance, with most studies only investigating distant effects on real-world performance (e.g., academic; Seery et al., 2010) or immediate effects on cognitive task performance (e.g., word finding; Mendes et al., 2007).

## Possible Underlying Mechanisms

Limited research has examined the potential mechanisms through which challenge and threat states influence performance (O'Connor, Arnold, & Maurizio, 2010). This is surprising, given the potential for such research to enhance theory and guide the development of theory-led interventions. Several underlying mechanisms have been proposed, including those related to emotions, attention, and physical functioning (Blascovich et al., 2004; Jones, Meijen, McCarthy, & Sheffield, 2009; Skinner & Brewer, 2004).

A challenge state is said to be associated with both positive and negative emotions, whereas a threat state is associated with only negative emotions (Jones et al., 2009; Skinner & Brewer, 2004). Furthermore, emotions are proposed to be interpreted as facilitative for performance in a challenge state but debilitative in a threat state (Jones et al., 2009; Skinner & Brewer, 2004). Recent research has supported this, demonstrating that a threat state is associated with greater cognitive and somatic anxiety and a more debilitative interpretation of anxiety responses compared to a challenge state (Quested et al., 2011; Williams, Cumming, & Balanos, 2010). Positive and negative emotions are typically associated with successful and unsuccessful performance, respectively, whereas facilitative interpretations of emotions predict more successful performance relative to debilitative interpretations (e.g., Nicholls, Polman, & Levy, 2012; Thomas, Maynard, & Hanton, 2007). A challenge state might therefore result in superior performance by promoting more favorable emotional responses (i.e., lower negative and higher positive emotions) and interpretation of emotions (i.e., more facilitative for performance).

A challenge state may also be associated with more effective attention compared to a threat state (Blascovich et al., 2004; Jones et al., 2009; Skinner & Brewer, 2004). During a challenge state the focus of attention is proposed to be on task-relevant cues, whereas in a threat state, attention is also directed to task-irrelevant cues (Jones et al., 2009). Research employing eye-tracking technology to objectively measure attention has demonstrated that efficient attention in aiming tasks is characterized by longer quiet eye durations (for a review, see Mann, Williams, Ward, & Janelle, 2007). The *quiet eye* is defined as the final fixation toward a relevant target prior to the initiation of a movement (Vickers, 2007). Longer quiet eye durations are proposed to extend a critical period of time during which task-relevant information gathered by preparatory fixations is processed and used to select, fine-tune, and program the motor response, resulting in more accurate performance (Mann, Coombes, Mousseau, & Janelle, 2011). Thus, a challenge state might result in better performance by encouraging more effective attentional control (i.e., longer quiet eye durations).

A small number of studies have shown that challenge and threat states lead to divergent behaviors or movements (O'Connor et al., 2010; Weisbuch, Seery, Ambady, & Blascovich, 2009). For example, Mendes and colleagues (2007) found that, compared to a challenge state, a threat state resulted in less effective movements during an interaction task, including greater freezing, avoidance posture, and less smiling. Thus, a challenge state might result in superior performance by encouraging task-related movement patterns that are more likely to translate to successful performance. Additionally, researchers have suggested that muscular tension is likely to be greater during a threat state than a challenge state (Wright & Kirby, 2003). To date, no studies have examined this proposition. However, given that lower muscle activity is typically associated with more successful performance (Lay, Sparrow, Hughes, & O'Dwyer, 2002), a challenge state might lead to better performance by encouraging lower activation of task-relevant muscles.

## The Present Study

The aim of the present study was to examine the influence of challenge and threat states on the performance of novice participants in a golf putting task and to identify the potential mechanisms through which these states operate (emotional, attentional, kinematic, and/or physiological). We predicted that the challenge group would display relatively higher cardiac output and lower total peripheral resistance compared to the threat group. Additionally, we predicted that the challenge group would perform better in the golf putting task than the threat group; display a more favorable emotional response (i.e., intensity and direction of cognitive and somatic anxiety); and display more effective attentional control (i.e., longer quiet eye durations), putting kinematics (i.e., lower clubhead acceleration and jerk), and muscle activity (i.e., lower extensor carpi radialis activity). Finally, to explore if differences in any of the process measures mediated any between-group differences in performance, mediation analyses were conducted (Hayes & Preacher, 2011).

## Method

### Participants

One hundred twenty-seven undergraduate students (63 women, 64 men) with a mean age of 19.47 years (*SD* = 2.48) participated in the study. All participants declared having no official golf handicap or prior formal golf putting experience and, thus, were considered novice golfers (Cooke, Kavussanu, McIntyre, & Ring, 2010; Moore, Vine, Cooke, Ring, & Wilson, 2012). Furthermore, all reported being right-handed, nonsmokers, free of illness or infection, having normal or corrected vision and no known family history of cardiovascular or respiratory disease, having not performed vigorous exercise or ingested alcohol for 24 h prior to testing, and having not consumed food or caffeine for 1 h prior to testing. Participants were tested individually. The protocol was approved by the local ethics committee, and written informed consent was obtained from each participant.

### Self-report Measures

#### Demand and resource evaluations

Demand and resource evaluations were assessed using the cognitive appraisal ratio (Tomaka, Blascovich, Kelsey, & Leitten, 1993). Demand evaluations were assessed by asking “How demanding do you expect the golf putting task to be?” and resource evaluations were assessed by asking “How able are you to cope with the demands of the golf putting task?” These two items were rated using a 6-point Likert scale anchored at 1 (*not at all*) and 6 (*extremely*). A ratio was then calculated by dividing demands by resources such that a value greater than 1 indicated a threat state and a value less than 1 indicated a challenge state. This self-report measure has been widely used in the challenge and threat literature (e.g., Feinberg & Aiello, 2010).

#### Cognitive and somatic state anxiety

The Immediate Anxiety Measurement Scale (IAMS; Thomas, Hanton, & Jones, 2002) was employed to assess the intensity and directional interpretation of anxiety symptoms experienced by participants. The IAMS provided definitions of cognitive and somatic anxiety, after which participants completed four items measuring the intensity and direction of each construct. The items were rated using a 7-point Likert scale anchored at 1 (*not at all*) and 7 (*extremely*) for intensity and −3 (*very negative*) and +3 (*very positive*) for direction. Thomas and colleagues (2002) provided evidence for the validity and reliability of this measure, and it has been used previously in the challenge and threat literature (e.g., Williams et al., 2010).

### Performance (Mean Radial Error)

Mean radial error (the average distance the ball finished from the hole in centimeters) was recorded as a measure of task performance. Zero was recorded and employed in the calculation of mean radial error on trials where the putt was holed (Cooke et al., 2010; Moore et al., 2012). Furthermore, on trials where the ball hit the boundary of the putting green (90 cm behind the hole) the largest error possible was recorded (90 cm). This occurred on 105 (14%) of the 762 trials (challenge = 32, threat = 73).

### Quiet Eye Duration

Gaze was measured using an Applied Science Laboratories (ASL; Bedford, MA) Mobile Eye Tracker. This lightweight system utilizes two features: the pupil and corneal reflection (determined by the reflection of an infrared light source from the surface of the cornea) to calculate point of gaze (at 30 Hz) relative to the eye and scene cameras mounted on a pair of spectacles. A circular cursor, representing 1° of visual angle with a 4.5-mm lens, indicating the location of gaze in a video image of the scene (spatial accuracy of ±0.5° visual angle; 0.1° precision), was viewed by the research assistant in real time on a laptop screen (Lenovo R500 ThinkPad) installed with *Eyevision* (ASL) recording software. Participants were connected to the laptop via a 10-m fire wire cable, and the researcher and laptop were located behind the participant to minimize distractions. The video data were recorded for subsequent off-line analysis.

The *quiet eye duration* was operationally defined as the final fixation toward the ball prior to the initiation of the backswing (Vickers, 2007). Quiet eye onset occurred before the backswing, and quiet eye offset occurred when the gaze deviated off the fixated object by 1° or more for more than 100 ms. A *fixation* was defined as a gaze maintained on an object within 1° of visual angle for a minimum of 100 ms (Moore et al., 2012). Each putt was subject to frame-by-frame video analysis using Quiet Eye Solutions software (http://www.QuietEyeSolutions.com). Unfortunately, gaze data for 21 participants (challenge = 10, threat = 11) could not be analyzed because of poor calibration. Thus, a total of 636 putts were analyzed. The researcher was blind to the test and status (group) of each participant when analyzing the data. A second analyst blindly scored 10% of the quiet eye duration data, and interrater reliability was assessed using the interobserver agreement method (Thomas & Nelson, 2001). This method estimates reliability using a formula that divides the number of commonly coded quiet eye durations (i.e., within 33.33 ms) by the sum of the commonly coded quiet eye durations and quiet eye durations coded differently. This analysis revealed a level of agreement at 81%.

### Cardiovascular Measures

A noninvasive impedance cardiograph device (Physioflow, PF05L1, Manatec Biomedical, Paris, France) was used to estimate heart rate, stroke volume, and cardiac output. The theoretical basis for this device and its validity during rest and exercise testing has been published previously (e.g., Charloux et al., 2000). The Physioflow measures impedance changes in response to a high-frequency (75 kHz) and low-amperage (3.8 mA) electrical current emitted via electrodes. Following preparation of the skin, six spot electrodes (Blue Sensor R, Ambu, Ballerup, Denmark) were positioned on the thorax: two on the supraclavicular fossa of the left lateral aspect of the neck, two near the xiphisternum at the midpoint of the thoracic region of the spine, one on the middle of the sternum, and one on the rib closest to V6. After the participant's details were entered (i.e., height, weight, etc.), the Physioflow was calibrated over 30 heart cycles while participants sat resting in an upright position. Three resting systolic and diastolic blood pressure values were taken (one prior to the 30 heart cycles, one during this time period, and another immediately after this time period) manually by a trained experimenter using an aneroid sphygmomanometer (ACCOSON, London, UK) and stethoscope (Master Classic II, Littmann, 3M Health Care, St. Paul, MN). The mean blood pressure values were entered into the Physioflow to complete the calibration procedure. Heart rate, stroke volume, and cardiac output were estimated continuously during baseline (5 min) and postmanipulation (1 min) time periods. Participants remained seated throughout these time periods. Reactivity, or the difference between the final minute of baseline and the minute postmanipulation, was examined for all cardiovascular variables.

Both heart rate and cardiac pre-ejection period are considered cardiovascular markers of task engagement, with greater increases in heart rate and greater decreases in cardiac pre-ejection period reflecting greater task engagement (Seery, 2011). The Physioflow does not allow for the computation of cardiac pre-ejection period, and so only heart rate was used in the present study to assess task engagement (as Derks, Scheepers, Van Laar, & Ellemers, 2011). Cardiac output and total peripheral resistance are cardiovascular indices that differentiate challenge and threat, with higher cardiac output and lower total peripheral resistance more reflective of a challenge state (Seery, 2011). Cardiac output was estimated directly by the Physioflow, and total peripheral resistance was calculated using the formula [mean arterial pressure × 80/cardiac output] (Sherwood et al., 1990). Mean arterial pressure was calculated using the formula [(2 × diastolic blood pressure) + systolic blood pressure/3] (Cywinski, 1980).

### Putting Kinematics

Acceleration of the clubhead in three axes was recorded using a tri-axial accelerometer (LIS3L06AL, ST Microelectronics, Geneva, Switzerland). Acceleration on the *X*, *Y*, and *Z* axes corresponded to lateral, vertical, and back-and-forth movement of the clubhead, which assessed clubhead orientation, clubhead height, and impact velocity, respectively. The signals were conditioned by a bespoke buffer amplifier with a frequency response of DC to 15 Hz. Both accelerometer and amplifier were mounted in a 39 mm × 20 mm × 15 mm plastic housing secured to the rear of the clubhead. A microphone (B5 Condenser, Behringer, Germany) connected to a mixing desk (Eurorack UB802, Behringer, Germany) was used to detect the putter–ball contact on each trial. These signals were digitized at 2500 Hz. A computer program determined clubhead kinematics for each putt from the onset of the foreswing phase of the putting stroke until the point of putter–ball contact. The average acceleration was calculated for the *X*, *Y*, and *Z* axes. Peak acceleration and root mean square jerk were also calculated for the *Z* axis as the primary axis involved in golf putting. The values from all trials were averaged to provide a test mean value for each kinematic variable (Cooke et al., 2010; Moore et al., 2012).

### Muscle Activity

Electromyographic activity of the extensor carpi radialis muscle of the left arm was recorded because previous research implicates this muscle as most influential in the golf putting stroke (Cooke et al., 2010; Moore et al., 2012). Muscle activity was measured using single-differential surface electrodes (DE 2.1, Delsys) and an amplifier (Bagnoli-4, Delsys) with a ground electrode on the collar bone. Electromyographic signals were amplified, filtered (20–450 Hz), and digitized (2500 Hz). The electromyographic signal for each trial was rectified, and the mean amplitudes (in microvolts) were calculated by averaging the activity over four consecutive periods: premovement initiation, backswing, foreswing, and postcontact. The duration of these periods was calculated from the *Z*-axis acceleration profile (described below). The backswing lasted from movement initiation until the top of the backswing; the duration of the premovement initiation was the same as the duration of the backswing. The foreswing lasted from the top of the backswing until putter–ball contact; the duration of the postcontact was the same as the duration of the foreswing. The trial values were averaged to provide a mean value for each electromyographic variable (Cooke et al., 2010; Moore et al., 2012).

### Procedure

First, participants were fitted with the physiological recording equipment and ASL Mobile eye tracker. Subsequently, 5 min of baseline cardiovascular data were recorded while participants sat still and quietly. Next, participants received their respective manipulation (challenge or threat; see Manipulation section). This was followed by a 1-min period during which cardiovascular data were recorded. Participants then completed the cognitive appraisal ratio and IAMS before performing six straight putts from three 1.83-m locations to a half-size hole (diameter = 6 cm) on an artificial putting green (length = 6 m, width = 2.5 m; Stimpmeter reading = 3.28 m). All participants used a standard length (90 cm) steel-shafted blade style golf putter (Sedona 2, Ping, Phoenix, AZ) and regular-size (diameter = 4.27 cm) white golf balls. Performance, gaze behavior, muscle activity, and kinematic data were continuously recorded throughout all putts. Finally, once the physiological recording equipment and ASL Mobile eye tracker had been removed, participants were thanked and debriefed about the aims of the study.

### Challenge and Threat Manipulations

Participants were randomly assigned to the two experimental groups. Challenge and threat states were manipulated through the instructional set given to participants. The instructions were adapted from previous research (e.g., Feinberg & Aiello, 2010; O'Connor et al., 2010). To foster task engagement, both groups received instructions emphasizing the importance of the task, that their score would be compared against others taking part (published leaderboard), that the task was going to be objectively evaluated (digital video camera), that low-performing participants would be interviewed, and that financial rewards existed for high-performing participants (top 5 performers awarded cash prizes of £50, £25, £20, £15, and £10, respectively). The threat instructions focused on the task's high degree of difficulty and emphasized that previous participants had struggled to perform well on the task. The challenge instructions focused on participants perceiving the task as a challenge to be met and overcome, thinking of themselves as capable of meeting that challenge, and emphasized that previous participants had performed well on the task (see the Appendix).

### Statistical Analysis

To ensure any between-group differences were not due to differences in gender, a series of independent *t* tests was conducted. These analyses revealed gender differences for cognitive appraisal ratio; cognitive anxiety direction; quiet eye duration; and muscle activity during the backswing, foreswing, and postcontact. Subsequently, one-way analyses of covariance (ANCOVAs) were conducted to examine between-group differences for these variables. The independent *t* tests revealed no gender differences for cognitive anxiety intensity, somatic anxiety intensity and direction, mean radial error, muscle activity pre-initiation, and all putting kinematic variables (*X*, *Y*, and *Z*-axis acceleration, peak acceleration, and root mean square jerk). Thus, a series of independent *t* tests was conducted on these variables to examine differences between the groups. Effect sizes were calculated using partial eta squared (ANCOVA) or Cohen's *d* (*t* test).

No gender differences existed for the cardiovascular variables. Task engagement was assessed using a dependent *t* test on the heart rate reactivity data to establish that, in the sample as a whole, heart rate increased significantly from baseline (i.e., heart rate reactivity greater than 0; as in Seery, Weisbuch, & Blascovich, 2009). Four univariate outliers (values more than 3.3 standard deviation units from the grand mean; Tabachnick & Fidell, 1996) from two participants were winsorized by changing the deviant raw score to a value 1% larger or smaller than the next most extreme score (as in Shimizu, Seery, Weisbuch, & Lupien, 2011). To differentiate challenge and threat states an index was created by converting each participant's cardiac output and total peripheral resistance residualized change scores into *z* scores and summing them. Residualized change scores were calculated in order to control for baseline values. Total peripheral resistance was assigned a weight of −1 and cardiac output a weight of +1, such that a larger value corresponded with greater challenge (as in Seery et al., 2009). To compare the groups, an independent *t* test was conducted on the challenge and threat index data.

Finally, to determine if significant differences in any of the process measures mediated any between-group differences in performance, mediation analyses were performed using the MEDIATE SPSS custom dialog developed by Hayes and Preacher (2011). This custom dialog tests the total, direct, and indirect effect of an independent variable on a dependent variable through a proposed mediator and allows inferences regarding indirect effects using percentile bootstrap confidence intervals.

## Results

### Manipulation Checks

The dependent *t* test on the heart rate reactivity data revealed that, in the sample as a whole, heart rate significantly increased from baseline, *t*(121) = 15.11, *p* < .001, *d* = 2.75, enabling the examination of challenge and threat states. The independent *t* test on the challenge and threat index data revealed a significant difference between the groups, *t*(120) = 2.63, *p* = .01, *d* = 0.48, with the challenge group (*M* = 0.45, *SD* = 2.05) exhibiting a larger index value than the threat group (*M* = −0.46, *SD* = 1.72). Furthermore, the one-way ANCOVA on the demand/resource evaluation data also revealed a significant difference between the groups, *F*(1,124) = 45.89, *p* < .001, 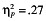, with the challenge group reporting a lower ratio score (*M* = 0.79, *SD* = 0.39) than the threat group (*M* = 1.39, *SD* = 0.62).

### Performance (Mean Radial Error)

The independent *t* test on the mean radial error data revealed a significant difference between the groups, *t*(125) = 3.84, *p* < .001, *d* = 0.69, with the challenge group (*M* = 35.48, *SD* = 14.82) achieving a lower mean radial error than the threat group (*M* = 46.53, *SD* = 17.45).

### Cognitive and Somatic Anxiety

The ANCOVA and independent *t* tests on the IAMS data revealed no significant difference between the groups in terms of the intensity of somatic anxiety, *t*(125) = 1.59, *p* = .12, *d* = 0.28, but significant differences between the groups in terms of the intensity of cognitive anxiety, *t*(125) = 2.86, *p* = .005, *d* = 0.51. The challenge group reported experiencing lower levels of cognitive anxiety than the threat group. Furthermore, these analyses revealed significant differences between the groups in terms of the direction of cognitive anxiety, *F*(1,124) = 18.38, *p* < .001, 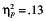, and somatic anxiety, *t*(125) = 2.45, *p* = .016, *d* = 0.44. Compared to the threat group, the challenge group interpreted the cognitive anxiety they experienced as more facilitative for their performance and the somatic anxiety they experienced as less debilitative. The cognitive and somatic anxiety data are presented in Table 1.

**Table 1:** Mean (*SD*) Emotional, Gaze, Putting Kinematic, and Muscle Activity Data for Challenge and Threat Groups

	Challenge		Threat	
		
	Mean	*SD*	Mean	*SD*
Cognitive anxiety intensity	3.05**	1.10	3.63	1.18
Cognitive anxiety direction	0.02***	1.14	−0.83	0.98
Somatic anxiety intensity	2.92	1.21	3.27	1.25
Somatic anxiety direction	−0.10*	1.07	−0.53	0.93
Quiet eye duration (ms)	1527.34*	814.28	1194.86	582.49
*X*-axis acceleration (m·s^−2^)	0.55**	0.25	0.69	0.33
*Y*-axis acceleration (m·s^−2^)	0.72*	0.20	0.83	0.31
*Z*-axis acceleration (m·s^−2^)	3.67**	1.12	4.33	1.26
Peak acceleration (m·s^−2^)	4.62***	1.31	5.48	1.58
Root mean square jerk (m·s^−2^)	3.71**	1.10	4.36	1.29
Pre-initiation muscle activity (μV)	15.12	7.39	17.98	15.36
Backswing muscle activity (μV)	22.36	13.92	25.60	18.56
Foreswing muscle activity (μV)	26.90*	17.93	34.63	22.50
Postcontact muscle activity (μV)	21.41*	11.07	28.61	19.72

*Note.* Significantly different from threat group, *p* < .05, **p < .01, ***p < .001.

### Quiet Eye Duration

The ANCOVA on the quiet eye duration data revealed a significant difference between the groups in terms of quiet eye duration, *F*(1,101) = 5.06, *p* = .027, 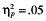. The challenge group displayed longer quiet eye durations than the threat group. The gaze data are presented in Table 1.

### Putting Kinematics

The independent *t* tests on the putting kinematic data revealed significant differences between the groups in terms of *X*-axis acceleration, *t*(124) = 2.68, *p* = .008, *d* = 0.48; *Y*-axis acceleration, *t*(124) = 2.38, *p* = .018, *d* = 0.43; *Z*-axis acceleration, *t*(124) = 3.08, *p* = .003, *d* = 0.55; peak acceleration, *t*(124) = 3.30, *p* < .001, *d* = 0.59; and root mean square jerk, *t*(124) = 3.02, *p* = .003, *d* = 0.54. The challenge group displayed lower lateral, vertical, and back-and-forth acceleration as well as lower peak acceleration and less root mean square jerk compared to the threat group. The putting kinematic data are presented in Table 1.

### Muscle Activity

The ANCOVA and independent *t* tests on the muscle activity data revealed no significant difference between the groups during pre-initiation, *t*(124) = 1.33, *p* = .19, *d* = 0.24, or the backswing, *F*(1,123) = 0.86, *p* = .36, 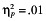, but a significant difference between the groups during the foreswing, *F*(1,123) = 3.72, *p* = .054, 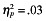, and post-contact, *F*(1,123) = 5.40, *p* = .022, 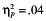. The challenge group exhibited less muscle activity during the foreswing phase and after putter–ball contact compared to the threat group. The muscle activity data are presented in Table 1.

### Mediation Analyses

To test if the effect of group on performance was mediated by any of the process variables, experimental group (coded challenge = 1, threat = 0) was entered as the independent variable, mean radial error was entered as the dependent variable, and a number of potential mediators were entered separately. Based on a 10,000 sampling rate, the results from bootstrapping revealed no significant indirect effects for cognitive anxiety intensity, 95% CI = −1.88 to 1.54; cognitive anxiety direction, 95% CI = −1.17 to 3.65; somatic anxiety intensity, 95% CI = −0.81 to 1.24; quiet eye duration, 95% CI = −2.09 to 1.53; pre-initiation muscle activity, 95% CI = −1.49 to 0.87; backswing muscle activity, 95% CI = −1.60 to 0.62; foreswing muscle activity, 95% CI = −3.11 to 0.21; or postcontact muscle activity, 95% CI = −2.84 to 0.48.

There were significant indirect effects for somatic anxiety direction, 95% CI = 0.01 to 3.45; *X*-axis acceleration, 95% CI = −6.39 to −0.88; *Y*-axis acceleration, 95% CI = −6.14 to −0.62; *Z*-axis acceleration, 95% CI = −5.20 to −0.71; peak acceleration, 95% CI = −5.97 to −0.83; and root mean square jerk, 95% CI = −5.15 to −0.70. Thus, multiple kinematic variables mediated the relationship between group and mean radial error. However, for somatic anxiety direction, the indirect (*b* = 1.42) and direct (*b* = −12.47) effects had opposite signs, and the direct effect was greater than the total (*b* = −11.05) effect. Thus, somatic anxiety direction had a suppression effect on the relationship between group and mean radial error (MacKinnon, Krull, & Lockwood, 2000). The mediation results are presented in Table 2.

**Table 2:** Mediation Results for All Emotional, Gaze, Putting Kinematic, and Muscle Activity Variables

	Effect	*SE*	LL 95% CI	UL 95% CI
Cognitive anxiety intensity	−0.17	0.82	−1.88	1.54
Cognitive anxiety direction	1.03	1.22	−1.17	3.65
Somatic anxiety intensity	0.15	0.48	−0.81	1.24
Somatic anxiety direction	1.42	0.89	0.01	3.45*
Quiet eye duration	−0.53	1.07	−2.90	1.53
*X*-axis acceleration	−3.50	1.41	−6.39	−0.88*
*Y*-axis acceleration	−3.28	1.43	−6.14	−0.62*
*Z*-axis acceleration	−2.62	1.15	−5.20	−0.71*
Peak acceleration	−3.00	1.31	−5.97	−0.83*
Root mean square jerk	−2.63	1.14	−5.15	−0.70*
Pre-initiation muscle activity	−0.28	0.57	−1.49	0.87
Backswing muscle activity	−0.25	0.54	−1.60	0.62
Foreswing muscle activity	−1.07	0.87	−3.11	0.21
Postcontact muscle activity	−1.13	0.85	−2.84	0.48

*Note.* LL: lower limit; CI: confidence interval; UL: upper limit.

* Significant indirect effect.

## Discussion

A challenge state has been associated with superior distant real-world performance compared to a threat state (Blascovich et al., 2004); however, no research has examined the immediate effect of these states on motor task performance. Furthermore, no research has examined the potential mechanisms through which these states might influence performance. Thus, the purpose of the present study was to investigate the immediate effect of challenge and threat states on the performance of novice participants in a golf putting task and examine multiple possible underlying processes.

### Challenge and Threat States and Performance

Consistent with previous research, challenge and threat states were manipulated via task instructions (e.g., Feinberg & Aiello, 2010; Tomaka, Blascovich, Kibler, & Ernst, 1997). The demand and resource evaluation data supported the effectiveness of the manipulation, as the challenge group reported a mean ratio score less than 1, reflecting a challenge state, and the threat group reported a mean ratio score greater than 1, reflecting a threat state. Thus, whereas the challenge group evaluated that they possessed the resources required to cope with the demands of the task, the threat group evaluated that they had insufficient resources to cope with the task demands. Several researchers have criticized self-report measures of challenge and threat states (e.g., Blascovich et al., 2004); therefore, the present study also adopted objective cardiovascular measures. Importantly, the heart rate data revealed that the whole sample was actively engaged in the task, as evidenced by increases in heart rate, allowing further examination of challenge and threat cardiovascular responses (Seery, 2011). The challenge and threat index data further supported the effectiveness of the manipulation, as the challenge group exhibited a larger index value, reflecting greater challenge (relatively higher cardiac output and lower total peripheral resistance; Seery, 2011) compared to the threat group.

As hypothesized, the performance data revealed that the challenge group performed better in the golf putting task than the threat group, achieving a lower mean radial error. This result equated to a medium to large effect size and is congruent with previous research showing that a challenge state is associated with higher levels of performance compared to a threat state (Gildea et al., 2007; Mendes et al., 2007; Seery et al., 2010). For example, Blascovich and colleagues (2004) demonstrated that experiencing a challenge state in response to a sport-relevant speech task was associated with superior real-world performance during the following season. The present study extends this research and is the first to demonstrate the immediate and direct effect (i.e., ∼2 min postmanipulation) of challenge and threat states on the performance of a novel motor task, with a challenge state resulting in superior motor task performance relative to a threat state. Given this finding, it is important to establish the underlying mechanisms through which these states influence performance, as such information may enhance theory and aid the design of effective theory-led interventions.

### Possible Underlying Mechanisms

The IAMS data revealed, as hypothesized, that challenge and threat states were associated with different emotional responses (see Table 1). There were no differences in terms of the intensity of somatic anxiety experienced; however, the challenge group reported experiencing lower levels of cognitive anxiety than the threat group. These findings are consistent with previous research demonstrating that a threat state is associated with greater cognitive anxiety (e.g., Quested et al., 2011). The IAMS data also revealed that the challenge group interpreted the cognitive anxiety they experienced as more facilitative for their performance and the somatic anxiety they experienced as less debilitative for their performance compared to the threat group. These findings are also congruent with previous research showing that a threat state is associated with a more debilitative interpretation of anxiety responses (e.g., Williams et al., 2010). Mediation analyses revealed a small suppression effect for somatic anxiety direction. Although a challenge state led to a more facilitative interpretation of somatic anxiety symptoms, this in turn led to poorer performance. This unexpected finding is inconsistent with our hypotheses and may be an artifact due to Type 1 error (MacKinnon et al., 2000). Future research should further investigate how challenge and threat states impact performance via emotional mechanisms.

Challenge and threat states were also associated with different movement patterns (see Table 1). The putting kinematic data revealed that, compared to the threat group, the challenge group displayed lower lateral, vertical, and back-and-forth clubhead acceleration as well as lower peak acceleration and less root mean square jerk. This movement pattern is more consistent with the movement pattern displayed by expert golfers (see Sim & Kim, 2010). The lower lateral (*X*-axis) acceleration suggests that the challenge group kept the clubhead more reliably aligned with the hole and avoided pushing or pulling putts, and the lower vertical (*Y*-axis) acceleration implies that the challenge group kept the clubhead more parallel to the ground and avoided imparting top or backspin on the ball. The lower back-and-forth (*Z*-axis) acceleration, peak acceleration, and root mean square jerk suggest that the challenge group performed with a smoother putting stroke and contacted the ball with less impact velocity, avoiding putts that were grossly overhit. Collectively, these findings support our hypotheses and add to previous research demonstrating that challenge and threat states can have divergent effects on movements (e.g., Mendes et al., 2007). Importantly, mediation analyses confirmed that all five of the putting kinematic variables mediated between-group differences in performance, suggesting that challenge and threat states predominantly impact upon performance by influencing the quality of task-related movements.

A challenge state is said to result in more effective attention compared to a threat state (Jones et al., 2009). The quiet eye duration data support this contention. As hypothesized, the challenge group displayed longer quiet eye durations than the threat group, a characteristic of more effective gaze behavior and attentional control in aiming tasks (Mann et al., 2007). By holding longer quiet eye durations on the ball, the challenge group may have extended the time in which the task-relevant information gathered by preparatory fixations was processed and used to select, fine-tune, and program the motor response (Mann et al., 2011). This may have increased the likelihood of correct decisions (e.g., distance to the hole) and accurate performance. However, mediation analysis revealed that quiet eye duration did not mediate between-group differences in performance. Thus, although challenge and threat states appear to differentially impact the efficiency of visual attentional control, these differences did not appear to significantly influence performance on the motor task.

It has been suggested that muscular tension is likely to be greater during a threat state than a challenge state (Wright & Kirby, 2003); however, to date, no studies have examined this proposition. The muscle activity data provide some support for this proposition. Although no differences in muscle activity existed between the groups prior to movement initiation or during the backswing, the challenge group exhibited lower extensor carpi radialis activity during the foreswing and after putter–ball contact compared to the threat group. Given that previous research has shown that lower activation of task-relevant muscles is associated with successful performance (e.g., Lay et al., 2002), the muscle activity pattern exhibited by the challenge group may be considered more effective for golf putting performance than the pattern exhibited by the threat group. Mediation analyses revealed that no muscle activity variable mediated between-group differences in performance. Therefore, although challenge and threat states appear to have divergent effects on muscle activity, these differences did not appear to impact upon task performance.

### Implications

The findings of the present study have some important implications. Specifically, from a theoretical perspective, the findings imply that the BPSM (Blascovich, 2008a) may provide a useful framework by which performance variability under stress can be examined. Furthermore, the findings suggest that interventions aimed at modifying the way in which individuals evaluate highly demanding and stressful tasks could significantly impact upon performance. Encouraging individuals to evaluate demanding tasks more adaptively, as a challenge rather than a threat, should facilitate more favorable emotional, attentional, kinematic, and physiological responses that ultimately benefit performance. Moreover, given that the cardiovascular response associated with a threat state is considered to have deleterious consequences for health when frequently experienced, such interventions may also have important health implications (Blascovich, 2008b).

A challenge state may be fostered by reducing the evaluated demands of the task or by increasing the actual or evaluated resources of the individual. Indeed, the findings of the present study and previous research suggest that such alterations could be made with an intervention as subtle and inexpensive as manipulating the way the task is framed (e.g., Feinberg & Aiello, 2010). Thus, coaches, managers, and sport psychologists should be mindful of the impact their instructions have on task performance and individuals' emotional, attentional, behavioral, and physiological responses. Tasks should be framed in a manner consistent with challenge, as this has the potential to lead to performance facilitation and more favorable responses.

### Limitations and Future Research

Despite the encouraging findings, the present study is not without its limitations. First, the adoption of a between-subjects design and the absence of a baseline performance condition may be viewed as potential limitations. However, previous challenge and threat research has successfully utilized a between-subjects design (e.g., Feinberg & Aiello, 2010). Furthermore, previous research has demonstrated that the amount of practice or exposure to a task dampens cardiovascular responses and that prior task performance has a significant impact on demand and resource evaluations (Kelsey et al., 1999; Quigley, Feldman Barrett, & Weinstein, 2002). Second, the use of multiple simple mediation analyses on many variables may be viewed as a potential limitation of the present study. Future research is therefore encouraged to develop and test more complex mediation models (e.g., challenge/threat → emotions → muscle activity → kinematics → performance) using statistical techniques such as structural equation modeling, although this would require a greater sample size than that in the present study to obtain adequate statistical power. The findings from such research are likely to substantially aid the development of theory and effective theory-based interventions.

Moreover, the fact that the present study only examined the effects of challenge and threat states over six trials may be viewed as a potential limitation. However, various researchers have noted the dynamic nature of demand and resource evaluations and how these evaluations tend to fluctuate during task performance as more information becomes available (Blascovich, 2008a; Jones et al., 2009; Quigley et al., 2002). Thus, whereas some individuals may begin by evaluating a task as a threat, this may change as early as after the first putt, and the task might become evaluated as less threatening or even challenging and vice versa. This reevaluation may have an impact on performance, and so the present study adopted a small number of trials to reduce the likelihood of reevaluation. Finally, the present study only examined the effects of challenge and threat states on individuals performing a novel motor task. Thus, the findings of the present study have limited generalizability. Future research should aim to investigate the effects of challenge and threat states on the performance of experienced individuals and whether the underlying mechanisms are consistent with those highlighted in the present study.

### Conclusion

The results demonstrate that challenge and threat states can have an immediate effect on motor task performance, with a challenge state resulting in superior performance relative to a threat state. Mediation analyses revealed that challenge and threat states influence performance via kinematic mechanisms, impacting the quality of task-related movements. The results highlight that the performance of a demanding and novel task can be facilitated by providing individuals with instructions that foster a challenge state, deemphasizing the difficulty of the task, and encouraging individuals to evaluate that they possess the resources required to cope with the task demands.

## Appendix: Challenge and Threat Manipulation Instructions

### Challenge Instructions

The rest period has now finished. We will shortly ask you to perform a golf putting task consisting of six golf putts from a distance of six feet. This is the most important part of the experiment and it is very important that you try, ideally, to get the ball in the hole or finish the ball as close to the hole as you possibly can with each putt. We will instruct you when you may hit each putt, and then you can hit each putt in your own time. After each putt, we will record the distance from the hole. Do you have any questions?

The mean distance from the hole will be calculated for each participant and placed on a leaderboard. At the end of the study the leaderboard will be emailed to all participants and displayed on a noticeboard. The top five performers will be awarded cash prizes of £50, £25, £20, £15, and £10, respectively. The worst five performers will be interviewed. Further, please note that each putt will be recorded on a digital video camera and may be used to aid teaching and presentations in the future.

Try and think of the upcoming golf putting task as a challenge to be met and overcome. Think of yourself as someone capable of meeting that challenge. We think that you are more than capable of meeting the challenges of the task. Our research has shown that most participants are able to handle tasks like the one you are about to complete. And although some participants may expect the task to be difficult, even participants with no or very limited golf putting experience have found that they are more than able to perform well on the task and have felt good about their performance. Again, although this task may sound difficult, remind yourself that you are capable of performing well and try your best.

With these instructions in mind, please now sit quietly for 1 minute and think about the upcoming task.

### Threat Instructions

The rest period has now finished. We will shortly ask you to perform a golf putting task consisting of six golf putts from a distance of six feet. This is the most important part of the experiment and it is very important that you try, ideally, to get the ball in the hole or finish the ball as close to the hole as you possibly can with each putt. We will instruct you when you may hit each putt, and then you can hit each putt in your own time. After each putt, we will record the distance from the hole. Do you have any questions?

The mean distance from the hole will be calculated for each participant and placed on a leaderboard. At the end of the study the leaderboard will be emailed to all participants and displayed on a noticeboard. The top five performers will be awarded cash prizes of £50, £25, £20, £15, and £10, respectively. The worst five performers will be interviewed. Further, please note that each putt will be recorded on a digital video camera and may be used to aid teaching and presentations in the future.

The upcoming golf putting task can be difficult and frustrating, and is a task you may not perform to a high standard. We think that you might struggle to meet the demands of the task. Our research has shown that most participants are unable to perform well on tasks like the one you are about to complete. Participants with no or limited golf putting experience may find the task difficult, and even expert golfers with extensive golf putting experience have found that they are unable to perform well on the task and have felt very unhappy about their performance. Again, although the task may sound difficult, do try your best.

With these instructions in mind, please now sit quietly for 1 minute and think about the upcoming task.
